# Miniaturized magnetic stir bars for controlled agitation of aqueous microdroplets

**DOI:** 10.1038/s41598-020-67767-z

**Published:** 2020-07-02

**Authors:** Pierre-Yves Gires, Mithun Thampi, Matthias Weiss

**Affiliations:** Experimental Physics I, University of Bayreuth, Universitätsstr. 30, 95447 Bayreuth, Germany

**Keywords:** Biophysics, Physical chemistry

## Abstract

Controlled stirring of tiny volumes of aqueous fluids is of particular importance in the life sciences, e.g. in the context of microfluidic and lab-on-chip applications. Local stirring not only accelerates fluid mixing and diffusion-limited processes, but it also allows for adding controlled active noise to the fluid. Here we report on the synthesis and characterization of magnetic nano-stir bars (MNBs) with which these features can be achieved in a straightforward fashion. We also demonstrate the applicability of MNBs to cell extract droplets in microfluidic channels and we show that they can introduce active noise to cell extracts as evidenced by altered fluctuations of ensembles of cytoskeletal filaments.

## Introduction

Major advances in the life sciences not only have fostered our understanding of living matter but also have prompted the development of a variety of new techniques and approaches in the physical sciences. Relating to diagnostic applications, for example, microfluidic devices have facilitated high-throughput screening for cells with an aberrant stiffness^1–4^ that is often associated with cancer. In the realm of synthetic biology, the demand for a controlled bottom-up production of small, cell-like entities and droplets also has driven the development of advanced microfluidic devices that support numerous lab-on-chip applications, e.g. studies on gene transcription in cell lysates^5,6^ and sorting of lysate droplets for directed evolution^7^. In this context, also a monitoring of the impact of mechanical and sterical constraints on self-organization events in cell extract droplets became possible, e.g. when reconstituting a mitotic spindle apparatus^8^. In fact, studying the dynamic self-organization and nonequilibrium thermodynamics of reconstituted biological systems via cell-like droplets has lately gained considerable momentum (see^9^ for a brief overview).

A potential limitation for studying dynamic emergent phenomena in crowded cellular extracts is, however, a slow or even anomalous diffusion^10,11^ that can severely impede important reactions even within small droplets. Therefore it would be desirable to be able to enhance mixing in microfluidic droplets. Besides this aspect, also the controlled addition of active noise would be beneficial, e.g. for probing fluctuation relations in non-equilibrium systems (see^12,13^ for recent reviews on this dynamically evolving topic).

So far, several approaches have been developed to enhance the mixing in microfluidic devices, e.g. a tuning of channel boundary conditions^14^, the application of acoustic waves^15^ or a laser beam^16^, and even first attempts to exploit a rotating electric or magnetic field that drives tiny stir bars^17,18^. Ensembles of tiny magnetic stir bars have recently also been shown to be capable of inducing macroscopic flow fields^19^. Indeed, rotating magnetic rods are particularly appealing since they can be easily adressed by an external magnetic field with negligible impedance issues (as compared to an application of acoustic or optical signals). Synthesizing small magnetic rods has been shown to be fairly straightforward when mimicking the natural synthesis of magnetosomes in magnetotactic bacteria^20^. Moreover, addressing an ensemble of magnetic rods by a rotary magnetic field shares many similarities with an ensemble of vortices, i.e. an ensemble of torque sources (rotlets). As a result, the collective dynamics can become chaotic as soon as four rotors interact^21^, which may eventually facilitate local fluid mixing.

Here, we report on the synthesis and characterization of magnetic nano-stir bars (MNBs) that can be included and activated in aqueous microdroplets, e.g. in microfluidic applications. In particular, we describe in some detail the production protocol and static properties of MNBs as well as their dynamics when addressing them with a rotary magnetic field. We also demonstrate the applicability of our MNBs to droplets of cell extract, produced in a microfluidic T-junction. As a signature of the active noise introduced by MNBs, we demonstrate that collections of cytoskeletal filaments in cell extract droplets display a markedly different undulation signature upon activating MNBs. To the best of our knowledge the latter application of MNBs has not been reported before.

## Nanobar fabrication and characterization

Putting an emphasis on materials and methods, this section provides details about the production of MNBs and the subsequent characterization of their shape and size.

### Nanobar synthesis

**Fig. 1:**
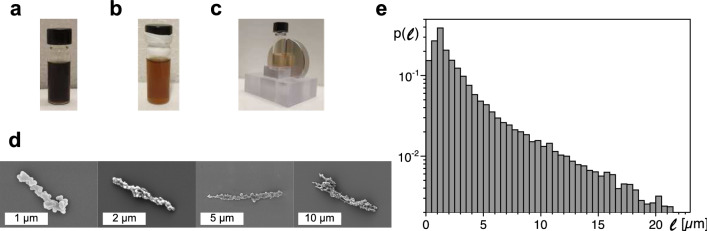
Suspension of Fe$_3$O$_4$ nanoparticles (**a**) at the beginning and (**b**) at the end of the ligand exchange process. (**c**) Alignment and synthesis of MNBs was performed in a custom-made holder with a neodymium magnet next to the vial. (**d**) Representative electron micrographs of individual MNBs (white scale bars indicate the stated lengths). (**e**) Length distribution, $p(\ell )$, obtained from seven different batches. Beyond a peak around $\ell \approx 1~\upmu$m a roughly exponential decay is observed (ensemble mean: $\langle \ell \rangle \approx 3.6~\upmu$m).

Starting point for the synthesis of nanobars was a suspension of spherical 30 nm oleic-acid stabilized iron oxide (Fe$_3$O$_4$) nanoparticles (NPs) dissolved in chloroform (SOR-30, Ocean NanoTech LLC). With a diameter of 30 nm, these ferrimagentic particles were well in the range for a single stable magnetic domain^22^. To make particles water-soluble, a ligand exchange reaction was performed. To this end, 20 μl of the NP suspension was mixed with 1.5 ml dimethylformamide, 1.5 ml dichlorobenzene, and 150 mg citric acid. The resulting black solution (Fig. 1a) was incubated at $100\,^\circ$C for 24 h and vortexed 4–5 times during this period to prevent clumping. The resulting brown solution (Fig. 1b) was centrifuged at 5,200*g* for 5 min to harvest citric-acid stabilized NPs. These hydrophilic NPs were dispersed in 1.5 ml deionized water and stored at $4\,^\circ$C.

For synthesizing MNBs a modified Stöber method^17^ was used, i.e. hydrophilic iron oxide NPs were first aligned in solution by a static magnetic field and subsequently connected permanently by silica coating. In particular, a solvent mixture of 400 μl deionized water and 1 ml isopropanol was added to 200 μl NP suspension, followed by 10 μl of tetraethylorthosilicate (after 2 min sonication) and 40 μl ammonia. The resulting solution was vortexed for 5 s and immediately placed next to a neodymium magnet in a custom-made holder to align nanoparticles into chains (see Fig. 1c). After incubating for 18 h, MNBs were concentrated at the wall of the vial next to the magnet. The magnet’s field strength (100–200 mT) was chosen sufficiently high to align nanoparticles and to orient their easy axes and magnetization parallel to the magnetic field^23^. Due to the silica matrix, these orientations were fixed during the synthesis, eventually yielding ferrimagnetic rods. Comparing the wobbling motion of individual rods (pinned with one end to a coverslip) to theoretical predictions^24^ additionally confirmed the ferrimagnetic character of MNBs.

After careful removal of the transparent supernatant, MNBs were redispersed in 1 ml of deionized water. Then, MNBs were purified by centrifuging five times at 2,500*g* for 6 min. Before the second centrifugation step the solution was vortexed for 5 s to remove loosely attached particles. Finally, the pellet was resuspended in 1 ml water and stored at $4\,^\circ$C.

### Nanobar shapes and length distribution

As a first step after synthesizing, we inspected the shape of MNBs by scanning electron microscopy (see Fig. 1d for representative electron micrographs). As a result, we observed that all MNBs had a rod-like shape with a slender appearance. In particular, we observed typical diameters in the range $\sim 300$ nm for MNBs in a length range from few 100 nm up to several micrometers. Longer MNBs appeared slightly more bulky (see Fig. 1d). To analyze the length distribution of MNBs, $p(\ell )$, we used transmission light microscopy. To this end, 0.5 μl of the suspension was dried on a plasma-cleaned coverslip and the resulting array of rods was imaged with an automatic tile scanning on an inverted microscope (Leica DMI6000B) equipped with a 20× objective (HC PL APO 20×/0.75 IMM CORR CS2) and a $1{,}392\times 1{,}040$ pixel camera (Leica DFC360FX), featuring a spatial resolution of 600 nm. Dark areas in the transmission images, corresponding to MNBs, were automatically detected via thresholding and elliptic fitting of connected regions with a custom-made Matlab code. The long axis of these ellipses was used as a measure for the MNB length, $\ell$. Accounting for the diffraction limit, all lengths below 600 nm were integrated into a single bin covering the range 0–500 nm.

The resulting probability density function of MNB lengths, $p(\ell )$, covered a length range from few hundred nanometers up to 20 μm, following roughly an exponential profile (see Fig. 1e). As expected for an expontial probability distribution, the mean length $\langle \ell \rangle \approx 4~\upmu$m was almost identical to the standard deviation ($\sigma (\ell )\approx \langle \ell \rangle$). Notably, different batches did not show significant deviations from each other. Thus, a large collection of slender MNBs with lengths $\ell <10~\upmu$m was reliably obtained with our synthesis approach.

### Rotation of nanobars upon driving with a magnetic field

**Fig. 2:**
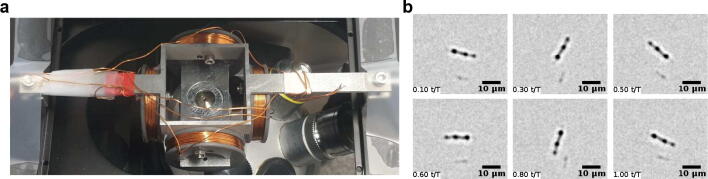
(**a**) Sample mounted on the stage of an inverted microscope: A microdroplet of the MNB solution, surrounded by squalene and sandwiched between two coverslips, was mounted on an aluminium holder and positioned in the center of two orthogonal Helmholtz coils; see also main text for details. (**b**) Representative snapshots of a single rotating MNB in an aqueous microdroplet when driving it with an alternating magnetic field ($f=0.1$ Hz); see supplementary movie 1 for the full time series.

Next, we aimed at exploring the dynamics of individual MNBs in aqueous droplets upon addressing them with a rotary magnetic field. To this end, we first diluted $8~\upmu$l of the MNB suspension in 1.5 ml water. Then, $0.4~\upmu$l of this solution was pipetted into a $4~\upmu$l squalene droplet between two 15 mm diameter coverslips, using a spacer made from four layers of magic tape (thickness $50~\upmu$m each) and one double side tape (thickness of $87.5~\upmu$m). The sandwiched sample was subsequently mounted on a custom-made aluminium holder that was then positioned at the intersection point of the axes of two orthogonally arranged, custom-made Helmholtz coils, similar to previous realizations^25^. Here, each coil consisted of 100 loops of copper wire with a diameter 0.5 mm, mounted on a holder with external diameter of 3 cm). The whole device was then mounted on the stage of the aformentioned inverted microscope (see Fig. 2a) and transmission light imaging was performed with a 10× objective (HC PL S-APO, 10×/2.3 DRY).

A custom-made microcontroller was used to operate the two pairs of Helmholtz coils, allowing us to create alternating magnetic fields in the frequency range $f\in [0,10]$ Hz with a tunable magnetic field strength (here: 0.5–0.9 mT). This field strength is well below the coercive field for magnetite^26^, i.e. the MNBs’ magnetization remained almost constant. When addressing individual MNBs in microdroplets in this way, they showed the anticipated rotation (see supplementary movie 1 and Fig. 2b for representative snapshots from this movie).

**Fig. 3:**
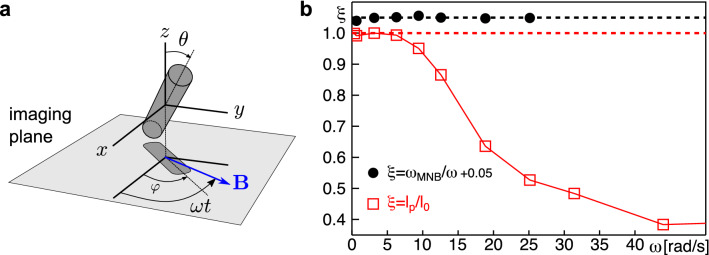
(**a**) Sketch of the coordinate system for evaluations: The magnetic field rotates with angular frequency $\omega$ in the *xy*-plane (the imaging plane), perpendicular to the optical axis (*z*-axis). Upon tilting, the MNB’s length, $\ell _0$, is projected to an apparent length $\ell _p=\ell _0\sin \theta$ in the imaging plane. The MNB’s angle with respect to the *x*-axis is denoted by $\varphi$. (**b**) For the representative case of a MNB with $\ell _0=16.6~\upmu$m we observed that the rod’s angular velocity, $\omega _{\text {MNB}}$, nicely followed the magnetic driving (black filled circles), i.e. the ratio $\xi =\omega _{\text {MNB}}/\omega$ was basically unity for a large range of driving frequencies, $\omega$ (these data have been shifted upwards by $+\,0.05$ for better visibility). Due to the finite image acquisition time (54 ms), determining $\omega _{\text {MNB}}$ for $\omega > 25~\text {rad/s}$ was not possible. In contrast, the projected length, $\ell _p$ only agreed with $\ell _0$ for small driving frequencies (open red squares), i.e. $\xi =\ell _p/\ell _0\approx 1$ was only observed for $\omega <5~\text {rad/s}$. For higher frequencies, the MNB was tilted with respect to the *xy*-plane and hence the time-averaged projected length was successively reduced.

For a quantitative evaluation, single MNBs were identified in transmission images as described before, and the orientation of the MNB with respect to the imaging coordinate system was retrieved. Here, the optical axis was chosen to be the *z*-axis of the coordinate system (see sketch in Fig. 3a) while the rotating magnetic quadrupole field coincided with the imaging plane (*xy*-plane). Driving single MNBs with different angular frequencies $\omega =2\pi f$ with $f\in [0.01,10]$ Hz, we extracted the projected length of the MNB perpendicular to the optical axis, $\ell _p=\ell _0\sin \theta$, and the angle $\varphi$ in the *xy*-plane from the recorded two-dimensional image time series.

As a result, we observed that the angular velocity of the MNB, $\omega _{\text {MNB}}=\Delta \varphi /\Delta t$, as extracted from the image time series, coincided perfectly with the angular frequency of the driving magnetic field, $\omega$ (see Fig. 3b for a representative example). The projected length $\ell _p$, however, was seen to match the actual MNB length $\ell _0$ only for low frequencies, whereas a progressive decrease started to emerge from $\omega \approx 5~\text {rad/s}$ for all analyzed MNBs (see Fig. 3b for an example). This finding indicates a tilting of the MNB out of the *xy*-plane, i.e. MNBs only act as a flat rotor for sufficiently small driving frequencies whereas a precession motion with a lower moment of inertia, phase-locked to the magnetic field, emerges for higher frequencies. In fact, the tilting is a generic effect for cylindrical rods for any slight misalignment between its symmetry axis and its magnetization^27^, and the resulting ‘wobbling motion’, has already been described before for magnetic rods^27,28^.

## Application of MNBs to microdroplets and microfluidics

Having synthesized and characterized MNBs, we next aimed at showing their compatibility with microfluidic devices and cell-derived fluids, both of which are commonly employed for lab-on-chip applications.

Resorting to a typical model system, we have chosen to mix MNBs into cell extract droplets derived from oocytes of *Xenopus laevis*. To this end, cytostatic factor arrested cytoplasmic extract was prepared from freshly laid eggs of *Xenopus laevis* following previously reported protocols^8,29^. In brief, eggs in metaphase of meiosis II were collected, dejellied, and crushed by centrifugation. The cytoplasmic layer was isolated, supplemented with an ATP-containing energy mix, and stored at $-\,80\,^\circ$C. Individual aliquots of extract were thawed prior to experiments and supplemented with MNBs. For this, $10~\upmu$l of MNB solution were centrifuged at 10,000*g* for 10 min, then the supernatant was gently removed and the MNB pellet was resuspended in $10~\upmu$l of cell extract by gentle flickering.

Microfluidic devices were produced by standard soft lithography: Digital masks for microfluidic T-junctions were designed with AutoCAD and transferred onto a spin-coated photoresist (SU-8 2050). To this end, a 50 μm thick film of SU-8 2050 was spin-coated on a 3 in.-diameter silicon wafer, soft-baked, and exposed using a MicroWriter ML3 (Durham MagnetoOptics). After post-exposure baking, the wafer was developed using SU-8 developer. PDMS (Sylgard elastomer 184) was mixed with curing agent in a 10:1 ratio and poured onto the wafer, degassed and cured at $75\,^\circ$C for 3.5 h. Then, the cured elastomer was removed from the wafer with a scalpel. Inlets and outlets were punched using a stainless steel puncher (Harris Uni-Core, 1.2 mm and 2 mm diameter, respectively). The device was then bonded to a microscope glass slide (cleaned by sonication in isopropanol for 10 min and dried in nitrogen flow) following a plasma exposure for 20 s at 916 mTorr. Bonding was strenghtened by storage at $75\,^\circ$C for one hour. Then the device was made hydrophobic by filling it with 2 μl of Aquapel rain repellent; the excess solvent was immediately blown out using nitrogen flow. Finally, the device (cross section 130 μm × 50 μm) was dried at $55\,^\circ$C to remove any residual solvent.

**Fig. 4:**
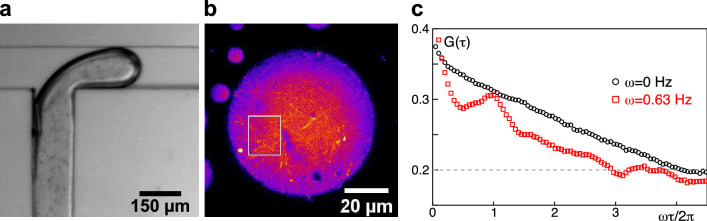
(**a**) Formation of cell extract droplets with immersed MNBs (seen as tiny black rods) at a microfluidic T-junction. For the full time series see supplementary movie 2. (**b**) Rectangular region of interest in a cell extract microdroplet with fluorescently labeled microtubules, used for calculating $G(\tau )$ as presented in subfigure c (see supplementary movies 4 and 5 for the associated time series). (**c**) The average Pearson correlation coefficient, $G(\tau )$ (defined in Eq. (1)) shows a monotonous and featureless decay for image time series of fluorescently labeled microtubules in a cell extract (black circles). Upon stirring with MNBs (red squares), these supposedly thermal undulations are supplemented with an active noise, leading to an overall faster decay in $G(\tau )$, but with oscillatory-like features that reflect the nearby MNBs’ tumbling motion.

Microdroplets of cell extracts with immersed MNBs ($10~\upmu$l starting material) were formed in an oil carrier fluid (squalene with cithrol DPHS at 50 mg/ml) at a microfluidic T-junction (see Fig. 4a and supplementary movie 2). Both fluids were introduced into the T-junction device by 1 ml glass syringes mounted on precision low-pressure syringe pumps (Cetoni). Connectics was made with PTFE tubing (1.58 mm outer diameter). Microdroplet formation was done at a flow rate of 50 $\upmu$l/h for both fluids. As can be seen in Fig. 4a (and in the associated supplementary movie 2), MNBs remained dispersed in the cell extract droplets formed at the T-junctions, i.e. they did not segregate to the interface region of both fluids. Furthermore, upon adressing MNBs again with an alternating magnetic field (amplitude 2 mT due to the fluid’s elevated viscosity) we observed the anticipated rotation (see representative case with $f=0.1$ Hz in supplementary movie 3). We infer from these observations that our MNBs are indeed compatible with microfluidic devices and can be used to stir cell extract microdroplets.

Finally we also wished to explore to which extent MNBs could introduce active noise to cell extract microdroplets. To this end we followed previous approaches to obtain polydisperse microdroplets with fluorescently labeled microtubules^8^ by adding rhodamine-labeled tubulin to cell extracts. Fluorescence imaging was performed on the same microscope as before, using a 63× oil immersion objective (HCX PL APO 63×/1.4), a 561 nm laser line (Coherent Sapphire), a 575–625 nm detection filter, and a spinning disk unit (Yokogawa, CSU X1) for confocal imaging. Images were taken with an EM-CCD camera (Roper Evolve 512, 512 × 512 pixel, 0.2 $\upmu$m per pixel, set to 16 bit mode) using a frame time $\Delta t=503$ ms.

While thermal noise and ATP/GTP-driven processes in the extract already induced some undulation and diffusion of these filaments (see supplementary movie 4), adding active noise by activating MNBs ($f=0.1$ Hz) resulted in marked changes (see supplementary movie 5). Due to the dense arrangement of filaments and other cell extract constituents, MNBs were frequently seen to perform a tumbling motion rather than rotating at constant angular velocity. For the purpose of adding active noise, however, the emergence of a less deterministic motion is supposedly even more beneficial.

To quantify the effect of this active stirring, we selected a region of interest with $n_p$ pixels that contained microtubules but not the nearby MNB (Fig. 4b). From the cropped image series *I*(*t*) we calculated the average correlation coefficient for a time lag $\tau =t_2-t_1$,

1 $$\begin{aligned} G(\tau )=\left\langle \frac{(I(t_1)-\langle I(t_1)\rangle )(I(t_2)-\langle I(t_2)\rangle )}{n_p\sqrt{\sigma ^2(I(t_1))\sigma ^2(I(t_2))}} \right\rangle _{t_1} \,\,, \end{aligned}$$

where $\sigma ^2(I)$ denotes the variance of all pixels’ fluorescence values at a given time. As can be seen in Fig. 4c, the signature of a non-stirred cell extract is a rather simple and featureless decay of $G(\tau )$. Upon stirring, however, an overall more rapid decay of $G(\tau )$ is observed, i.e. correlations in the filaments’ motion are partially destroyed by the active noise introduced by the nearby MNB. On top of this, an almost oscillatory modulation, reflecting the MNB’s tumbling, is observed. Hence, microtubules are locally shaken by the action of nearby MNBs, i.e. biologically active droplets can indeed be equipped with externally controlled active noise via MNBs. We anticipate that this feature will be a versatile tool for future work on non-equilibrium fluctuations in biological or artificial fluids.

## Conclusion

We have reported here in some detail on the synthesis and characterization of magnetic nano-stir bars (MNBs). Following our protocols, the resulting MNBs assumed slender shapes with typical diameters in the range of few hundred nanometers and a length range from few hundred nanometers to about 20 μm. These MNBs can be addressed and controlled well by an alternating magnetic quadrupole field, leading to a rotation of MNBs in aqueous fluids. Application of MNBs to microfluidic devices was demonstrated and the introduction of active noise to cell extract droplets was highlighted by quantifying temporal correlations of ensembles of cytoskeletal filaments. In fact, we are not aware of any previous study that has used MNBs to agitate cell extracts in a controlled fashion and quantitfy the resulting effects on fluctuations. Based on our data, we believe that such applications of MNBs can become versatile tools, e.g. in the context of lab-on-chip developments and for studying tiny volumes of biologically active fluids.

## Supplementary information

Supplementary file1

Supplementary file2

Supplementary file3

Supplementary file4

Supplementary file5

## Data Availability

Datasets generated and analyzed during the current study are available from the leading author upon reasonable request (Pierre-Yves.Gires@uni-bayreuth.de).
